# Diagnostic Accuracy of Coronary Angiography-Based Vessel Fractional Flow Reserve (vFFR) Virtual Stenting

**DOI:** 10.3390/jcm11051397

**Published:** 2022-03-03

**Authors:** Mariusz Tomaniak, Tara Neleman, Anniek Ziedses des Plantes, Kaneshka Masdjedi, Laurens J. C. van Zandvoort, Janusz Kochman, Wijnand K. den Dekker, Jeroen M. Wilschut, Roberto Diletti, Isabella Kardys, Felix Zijlstra, Nicolas M. Van Mieghem, Joost Daemen

**Affiliations:** 1 Department of Cardiology, Erasmus University Medical Center, ThoraxCenter, 3000 CA Rotterdam, The Netherlands; mariusz.tomaniak@wum.edu.pl (M.T.); t.neleman@erasmusmc.nl (T.N.); a.ziedsesdesplantes@erasmusmc.nl (A.Z.d.P.); k.masdjedi@erasmusmc.nl (K.M.); l.vanzandvoort@erasmusmc.nl (L.J.C.v.Z.); w.dendekker@erasmusmc.nl (W.K.d.D.); j.wilschut@erasmusmc.nl (J.M.W.); r.diletti@erasmusmc.nl (R.D.); i.kardys@erasmusmc.nl (I.K.); f.zijlstra.1@erasmusmc.nl (F.Z.); n.vanmieghem@erasmusmc.nl (N.M.V.M.); 2 First Department of Cardiology, Medical University of Warsaw, 02-091 Warsaw, Poland; jkochman@wum.edu.pl

**Keywords:** angiography-based FFR, fractional flow reserve, percutaneous coronary intervention, vFFR, computational fluid dynamics, coronary artery disease, residual ischemia, virtual PCI, novel interventional coronary diagnostics

## Abstract

3D coronary angiography-based vessel fractional flow reserve (vFFR) proved to be an accurate diagnostic alternative to invasively measured pressure wire based fractional flow reserve (FFR). The ability to compute post-PCI vFFR using pre-PCI vFFR virtual stent analysis is unknown. We aimed to assess the feasibility and diagnostic accuracy of pre-PCI vFFR virtual stenting analysis (residual vFFR) with post-PCI FFR as a reference. This is an observational, single-center retrospective cohort study including consecutive patients from the FFR-SEARCH registry. We blindly calculated residual vFFR from pre-PCI angiograms and compared them to invasive pressure-wire based post-PCI FFR. Inclusion criteria involved presentation with either stable or unstable angina or non-ST elevation myocardial infarction (NSTEMI), ≥1 significant stenosis in one of the epicardial coronary arteries (percentage diameter stenosis of >70% by QCA or hemodynamically relevant stenosis with FFR ≤0.80) and pre procedural angiograms eligible for vFFR analysis. Exclusion criteria comprised patients with ST elevation myocardial infarction (STEMI), coronary bypass grafts, cardiogenic shock or severe hemodynamic instability. Eighty-one pre-PCI residual vFFR measurements were compared to post-PCI FFR and post-PCI vFFR measurements. Mean residual vFFR was 0.91 ± 0.06, mean post-PCI FFR 0.91 ± 0.06 and mean post-PCI vFFR was 0.92 ± 0.05. Residual vFFR showed a high linear correlation (r = 0.84) and good agreement (mean difference (95% confidence interval): 0.005 (−0.002–0.012)) with post-PCI FFR, as well as with post-PCI-vFFR (r = 0.77, mean difference −0.007 (−0.015–0.0003)). Residual vFFR showed good accuracy in the identification of lesions with post-PCI FFR < 0.90 (sensitivity 94%, specificity 71%, area under the curve (AUC) 0.93 (95% CI: 0.86–0.99), *p* < 0.001). Virtual stenting using vFFR provided an accurate estimation of post-PCI FFR and post-PCI vFFR. Further studies are needed to prospectively validate a vFFR-guided PCI strategy.

## 1. Introduction

Functional physiological lesion assessment after angiographically successful percutaneous coronary intervention (PCI) proved to have significant prognostic value [1,2,3,4,5,6,7,8,9,10]. More specifically, individuals with higher post-PCI fractional flow reserve (FFR) values had improved prognosis [8,10,11,12,13,14,15].

Vessel fractional flow reserve (vFFR) has been recently introduced into the armamentarium of catheterization laboratory practice aiming to simplify functional lesion assessment [5,16]. Its good correlation to invasive FFR, both in a pre- and post-PCI setting, was recently demonstrated [17,18,19,20,21,22,23,24,25,26]. vFFR allows computation of FFR using a 3D reconstruction of coronary angiography without the necessity for a pressure wire or hyperemic agent [16,18].

As a next step, the ability to predict the functional outcomes of PCI is gaining increased attention [27,28,29,30]. Recent developments in the vFFR software allowed us to simulate the effect of ‘virtual’ PCI and estimate post-PCI FFR (residual vFFR). As of to date, the diagnostic performance of residual vFFR assessment using baseline angiograms has not been evaluated.

Here, we present the ‘Virtual stenting’ vFFR study that aimed to assess the diagnostic performance of residual vFFR (‘virtual stenting’ vFFR)—performed using the pre-PCI angiogram—against invasively measured post stenting FFR in a consecutive series of patients.

## 2. Materials and Methods

The ‘Virtual stenting’ vFFR study is an observational, single-center cohort study aiming to evaluate the diagnostic performance of offline pre-PCI angiogram-based estimation of final functional PCI outcome—namely residual vFFR—against invasively measured post-PCI FFR. A consecutive cohort of the 200 most recent patients enrolled in the FFR SEARCH registry who underwent PCI with stenting were screened for eligibility [31,32]. The detailed study design and protocol of FFR SEARCH registry have been previously described [31]. In brief, FFR SEARCH was a prospective registry in which FFR values were routinely collected after successful PCI in 1000 consecutive patients between March 2016 and May 2017. Inclusion criteria in the present study were age ≥18 years, presentation with either stable or unstable angina or non-ST elevation myocardial infarction (NSTEMI), at least one significant stenosis in one of the epicardial coronary arteries (diameter stenosis of >70% by QCA or hemodynamically significant stenosis defined as FFR ≤ 0.80). Exclusion criteria comprised of ST elevation myocardial infarction (STEMI), coronary bypass grafts (CABG), ostial lesions, cardiogenic shock or severe hemodynamic instability, or adenosine intolerance. In addition, patients with inadequate pressure waveform or lack of two adequate orthogonal views to create a 3D reconstruction of the target artery pre- and post-PCI to subsequently calculate a vFFR value were excluded.

The study was conducted in accordance with the Declaration of Helsinki. The study protocol was approved by the Erasmus University Medical Center ethics committee (MEC-2016-063). All patients provided written informed consent for the procedure and the use of anonymous datasets for research purposes in alignment with the Dutch Medical Research Act.

### 2.1. FFR and Angiogram Acquisition

FFR was measured using a dedicated microcatheter (Navvus RXi™, ACIST Medical Systems, Eden Prairie, MN, USA). FFR was defined as the mean distal coronary artery pressure divided by mean aortic pressure during maximum hyperemia induced by continuous intravenous infusion of adenosine at a rate of 140 μg kg^−1^·min^−1^ through an antecubital vein [32]. Post-PCI FFR assessment was completed at 2 cm distal from the most distal stent edge [32]. Subsequently, two standard monoplane angiographic views (≥30° apart, preferably orthogonal) were collected after a bolus of 200 mcg nitroglycine. For each patient, the position of the Navvus catheter was recorded. Aortic root pressure was continuously recorded, the pressure measurement registered before the start of the FFR measurement was utilized as input in the vFFR software [18].

Pre- and post-PCI vFFR analysis was conducted offline by trained analysts blinded to the invasive FFR values using dedicated software (CAAS workstation 8.3 software, Pie Medical Imaging, Maastricht, The Netherlands). The methodology of pre- and post-PCI vFFR calculation has been reported in detail elsewhere [18,24,26].

Pre- and post-PCI angiograms were used to compute pre- and post-PCI vFFR, respectively. Apart from two angiographic views with ≥30° differences in rotation/angulation to produce a 3D reconstruction of the coronary artery, also the views demonstrating the position of FFR microcatheter were checked upon post-PCI vFFR measurement. All analyses were blinded to the invasive FFR value.

Whereas the alignment of the cardiac cycle between the two angiograms was performed automatically based on ECG triggering, manual frame selection was possible [18]. Vessel contour delineation was completed semi-automatically from the ostium to the most distal position of the Navvus catheter. The percent diameter stenosis, minimal lumen diameter, reference lumen diameter, minimal lumen area, and lesion length were determined from the same 3D model as in which the vFFR was estimated [18].

### 2.2. ‘Virtual Stenting’ (Residual) vFFR Analysis

**Figure 1:**
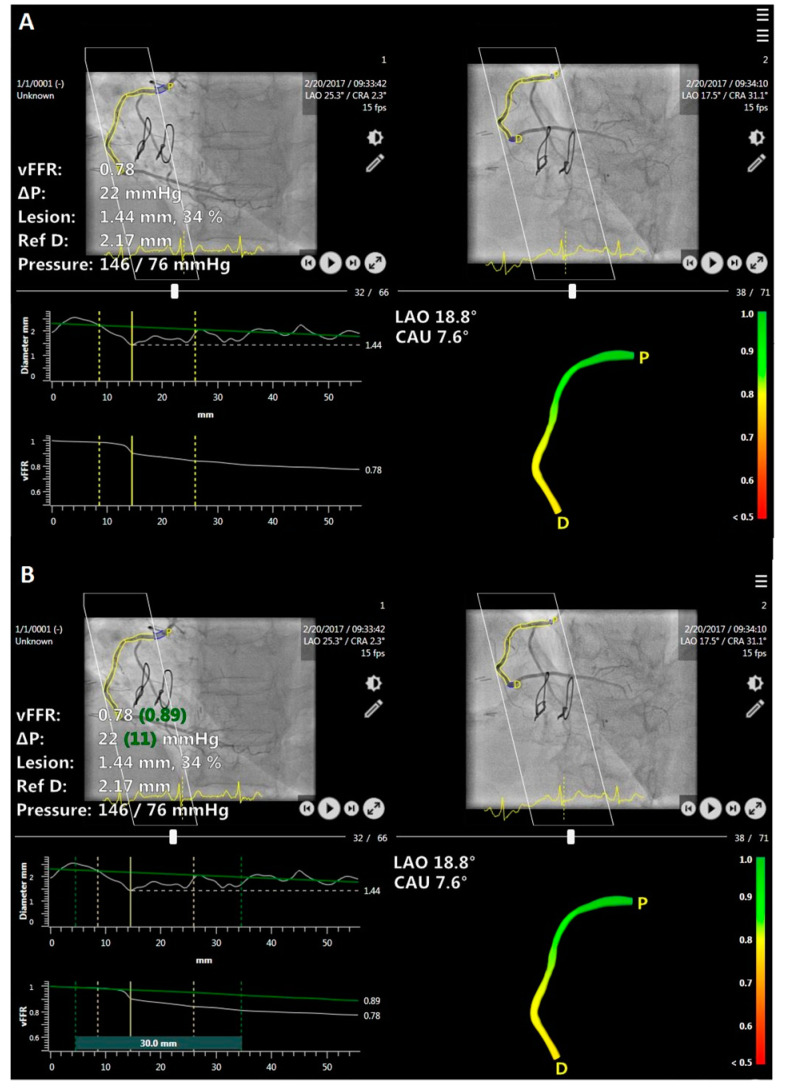
Computation of vessel fractional flow reserve (vFFR) (value in white font) (**A**) and virtual stenting vFFR (value in green font) (**B**) based on two pre-percutaneous coronary intervention (PCI) angiograms. Δ pressure—change in the pressure, Ref D—reference diameter, P—proximal, D—distal, LAO—left anterior oblique, CAU—caudal.

The concept of residual vFFR (‘virtual stenting’ vFFR) involves computation of vFFR assuming that the diseased segment of the coronary artery has been treated with a successful stent implantation eliminating the drop of vFFR in the selected artery segment (Figure 1). Residual vFFR was calculated using the pre-PCI angiograms by analysts blinded to invasive FFR and post-PCI vFFR measurements. No manual adjustment for the estimated target stent length or location was performed in the present investigation, in which all the analyzed residual vFFR values were generated automatically by the software.

Correlations between residual vFFR and post-PCI FFR and between residual vFFR and post-PCI vFFR were evaluated.

In addition, the diagnostic accuracy of residual vFFR to identify post-PCI FFR <0.90 was assessed. The threshold of <0.90 for post-PCI FFR has been selected based on prior reports suggesting more favorable clinical outcomes in case the post PCI FFR values exceeded this threshold [8,11].

### 2.3. Statistical Analysis

Variable distributions were evaluated by Kolmogorov–Smirnov tests. Normally distributed continuous variables are displayed as mean ± standard deviation (SD), and compared using the Student’s *t*-test. Continuous variables with non-normal distribution are presented as median (25th–75th percentile), and compared using the Mann–Whitney test. Categorical variables are shown as counts and percentages and compared with the use of chi-square or Fisher exact tests. The correlations between residual vFFR and post-PCI FFR and post-PCI vFFR were evaluated using the Pearson R or Spearman’s rank correlation coefficients, for variables with normal and non-normal distribution, respectively. Agreement between the indices was evaluated by Bland–Altman plots depicting mean differences and corresponding 95% limits of agreement.

Receiver–operating curves (ROC) were used to assess the discriminative ability of the residual vFFR to detect a post-PCI microcatheter-based FFR < 0.9 which has been used in previous studies as a cut-off value to predict clinical outcome [8,12,13]. Statistical analyses were performed using the SPSS statistical package version 24 (IBM, Armonk, North Castle, NY, USA).

## 3. Results

A total of 200 patients who underwent post-PCI evaluation with invasive FFR were screened. Residual ‘virtual stenting’ vFFR and post-PCI vFFR computation were subsequently performed in 81 eligible individuals. Key reasons for screening failure included: presentation with STEMI (*n* = 90), lack of two sufficient orthogonal angiographic views > 30 degrees or substantial overlap/foreshortening in pre-PCI (*n* = 19) or post-PCI (*n* = 21) angiograms, inadequate pressure waveforms (*n* = 10) or assessment of bypass grafts (*n* = 9). Baseline clinical characteristics are summarized in Table 1.

Mean age was 64 ± 11 years, 48 (59.3%) were male. Diabetes was present in 20 (24.7%) of the patients. A history of previous myocardial infarction (MI) or prior PCI was present in 18.5% and 30.9% of the patients, respectively. In 49.4% of the patients, the FFR measurement was performed in the left anterior descending artery. Mean 3D QCA-based diameter stenosis pre-PCI was 53 ± 15% with a reference vessel diameter of 2.90 ± 0.65 mm (Table 2). Mean pre-PCI vFFR was 0.72 ± 0.17.

**Figure 2:**
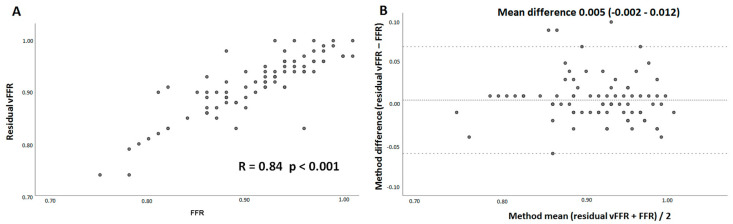
Scatter plot demonstrating the association between residual vessel-FFR (virtual stenting vFFR) and invasively measured post-PCI FFR (**A**) and Bland–Altman plots of differences versus the means (**B**). The mean difference and the 95% confidence interval are presented. vFFR, vessel fractional flow reserve, FFR, fractional flow reserve.

**Figure 3:**
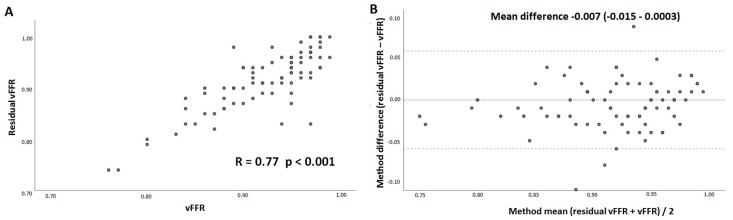
Scatter plot demonstrating the association between residual vessel-FFR (virtual stenting vFFR) and post-PCI vFFR (**A**) and Bland–Altman plots of differences versus the means (**B**). The mean difference and the 95% confidence interval are presented. vFFR, vessel fractional flow reserve.

There were no significant differences observed between estimated mean residual vFFR (0.91 ± 0.06) and actual post-PCI FFR value (0.91 ± 0.06). Likewise, no differences were found between estimated residual vFFR (0.91 ± 0.06) and post-PCI vFFR (0.92 ± 0.05). A good linear correlation was observed between residual vFFR and post-PCI FFR (r = 0.84, *p* < 0.001) as well as between residual vFFR and post-PCI vFFR (r = 0.77, *p* < 0.001) (Figure 2A and Figure 3A). Bland Altman analyses, expressed as the difference between residual vFFR and post-PCI FFR versus their average, showed a mean difference (95% CI) of 0.005 (−0.002–0.012) (Figure 2B), whereas the difference between the residual vFFR and post-PCI vFFR versus their average, showed a mean difference (95% CI) of −0.007 (−0.015–0.0003) (Figure 3B).

**Figure 4:**
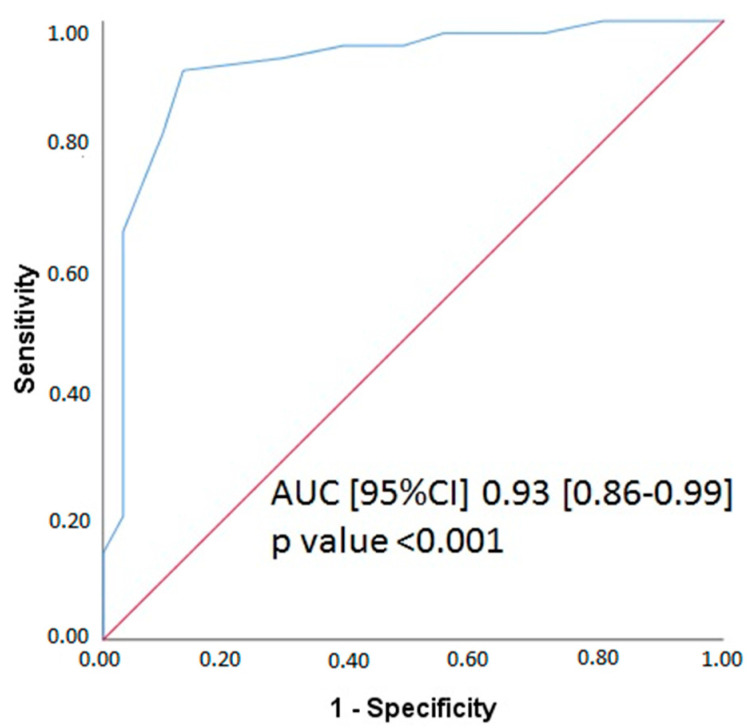
ROC curve for residual vessel FFR (virtual stenting vFFR). Comparison is made with an FFR at a cut point of <0.90. vFFR, vessel fractional flow reserve, FFR—fractional flow reserve, AUC—area under the curve, 95% CI—95% confidence interval.

There were 31 lesions (38.5%) identified with post-PCI FFR < 0.90. Residual vFFR showed a good accuracy in the identification of lesions with post-PCI FFR < 0.90 (sensitivity 94%, specificity 71%, area under the curve (AUC) 0.93 (95% CI: 0.86–0.99), *p* < 0.001) (Figure 4).

## 4. Discussion

The present study is the first to evaluate the feasibility of vFFR estimation of post PCI functional outcome. Residual vFFR calculated based on pre-PCI angiograms simulating the effects of coronary stent implantation correlates well with both post-PCI FFR and post-PCI vFFR values. Moreover, discriminative ability for post-PCI FFR < 0.9 was good, without the necessity for an invasive pressure wire or microcatheter and pharmacological induction of hyperemia, before actual stent implantation in patients presenting with either chronic coronary syndrome or non-ST-ACS.

Indeed, a considerable proportion of patients after successful PCI still suffer from angina [7]. The latter has been linked to functionally unresolved ischemia, which might in turn be the underlying cause of adverse prognosis [7,9,10,11]. As such, the assessment of post PCI FFR proved to be an effective metric to quantify residual ischemia [1,2,3,4,5,6,7,8,9,10,11].

The presented findings, although preliminary, constitute another step forward in the development of virtual PCI planning tools. Our study extends prior observations from computed tomography coronary angiography (CCTA) based FFRCT PCI planning software [27] and other angiography-derived residual FFR indices [25,28,29,30]. These tools have the potential to improve treatment qualification by better predicting which patients end with proper functional PCI outcomes as well as those with a lower likelihood of functionally satisfactory outcome, and thus, a risk of a futile invasive procedure. Moreover, clinical application of pre-PCI estimation of post PCI residual vFFR value could allow the interventionalist to anticipate the possible post procedural functional outcome before proceeding with actual stenting, which could help to develop a precise, individual patient-specific revascularization strategy [27]. Of note, reliable derivation of angiography-based FFR indices might also be feasible using the contemporary low X-ray frame and pulse rate settings [33]. Nevertheless, a recent phantom study addressing the importance of 3D-QCA reconstruction accuracy when computing virtual FFR from invasive angiography identified some small reconstruction errors further emphasizing the need for optimal quality angiogram acquisition for vFFR computation [34].

Importantly, virtual stenting vFFR cannot account for heavy calcifications or stent underexpansion; the software used in this study assumes an almost perfect PCI result.

Virtual stenting vFFR predicts the physiological response to PCI and is not intended to be a replacement for optical coherence tomography or intravascular ultrasonography in determining procedural success, which is dependent on several procedural factors [35]. As such, the ‘residual’ vFFR could be perceived as a complementary rather than individual stand-alone diagnostic modality. It is of note, however, that residual vFFR was able to predict most (94%) of the patients with post PCI FFR < 0.90.

### Study Limitations

The following limitations of this study need to be noted while interpreting its results. This is a retrospective cohort study with a relatively small sample size. The residual vFFR was compared with post PCI FFR analyzed with a dedicated microcatheter known to slightly overestimate FFR values [36,37]. Importantly, the angiographic criteria had to be fulfilled both for pre- and post-PCI angiograms in our study, resulting in a considerable proportion of non-eligible patients. The significant percentage of patients excluded due to nonanalyzable angiograms should also be put into perspective for procedures that were done in routine cathlab practice with a lack of intention for concomitant pre and post PCI vFFR analysis. Finally, the effectiveness and potential superiority of the use of residual vFFR to guide stent implantation in routine clinical practice remains to be established.

## 5. Conclusions

Pre-PCI estimation of ‘residual’ vFFR based upon invasive angiographic imaging is feasible, correlates well with post PCI invasive FFR and vFFR measurements and can predict physiological response to stenting with high accuracy. Further studies are needed to evaluate the efficacy and safety of 3D-QCA based FFR-guided coronary interventions.

## Figures and Tables

**Table 1:** Baseline characteristics (patients with angiograms analyzable both pre and post PCI).

	*n* = 81
Age	64.0 ± 11.0
Male	48 (59.3%)
BMI	27.1 ± 4.6
Diabetes	20 (24.7%)
Hypertension	47 (58.0%)
Dyslipidaemia	42 (51.9%)
Prior PCI	25 (30.9%)
Prior MI	15 (18.5%)
Prior stroke	15 (18.5%)
Peripheral artery disease	11 (13.5%)
Current smoker	25 (30.9%)
Creatinine (mmol/dl)	90.8 ± 31.0

BMI—body mass index, PCI—percutaneous coronary intervention, MI—myocardial infarction Data presented as count (*n*) and percentages or mean ± standard deviation (SD).

**Table 2:** Pre-PCI lesion and procedural characteristics.

	*n* = 81
Measured artery	
Left main coronary artery	2 (2.4)
Left anterior descending	40 (49.4)
Left circumflex	20 (24.7)
Right coronary artery	19 (23.5)
American College of Cardiology (ACC)/American Heart Associations (AHA) lesion type	
A	10 (12.3)
B1	21 (25.9)
B2	22 (27.2)
C	28 (34.6)
Bifurcation	10 (12.3%)
Calcification	64.0 ± 11.0
Three-dimensional-quantitative coronary angiography (QCA) analyses	
Diameter stenosis (%)	53 ± 15
Minimal lumen diameter, mm	1.39 ± 0.96
Lesion length, mm	17 ± 9
Reference diameter, mm	2.90 ± 0.65
Percutaneous coronary intervention (PCI) Procedure	
Predilatation	52 (64.2%)
Number of stents implanted	1.44 ± 0.67
Postdilatation	62 (76.5%)

Data presented as count (*n*) and percentages or mean ± standard deviation (SD) LM—left main coronary artery, LAD—left anterior descending, Cx—left circumflex, RCA—right coronary artery.

## Data Availability

Data available on request due to restrictions (privacy or ethical).
